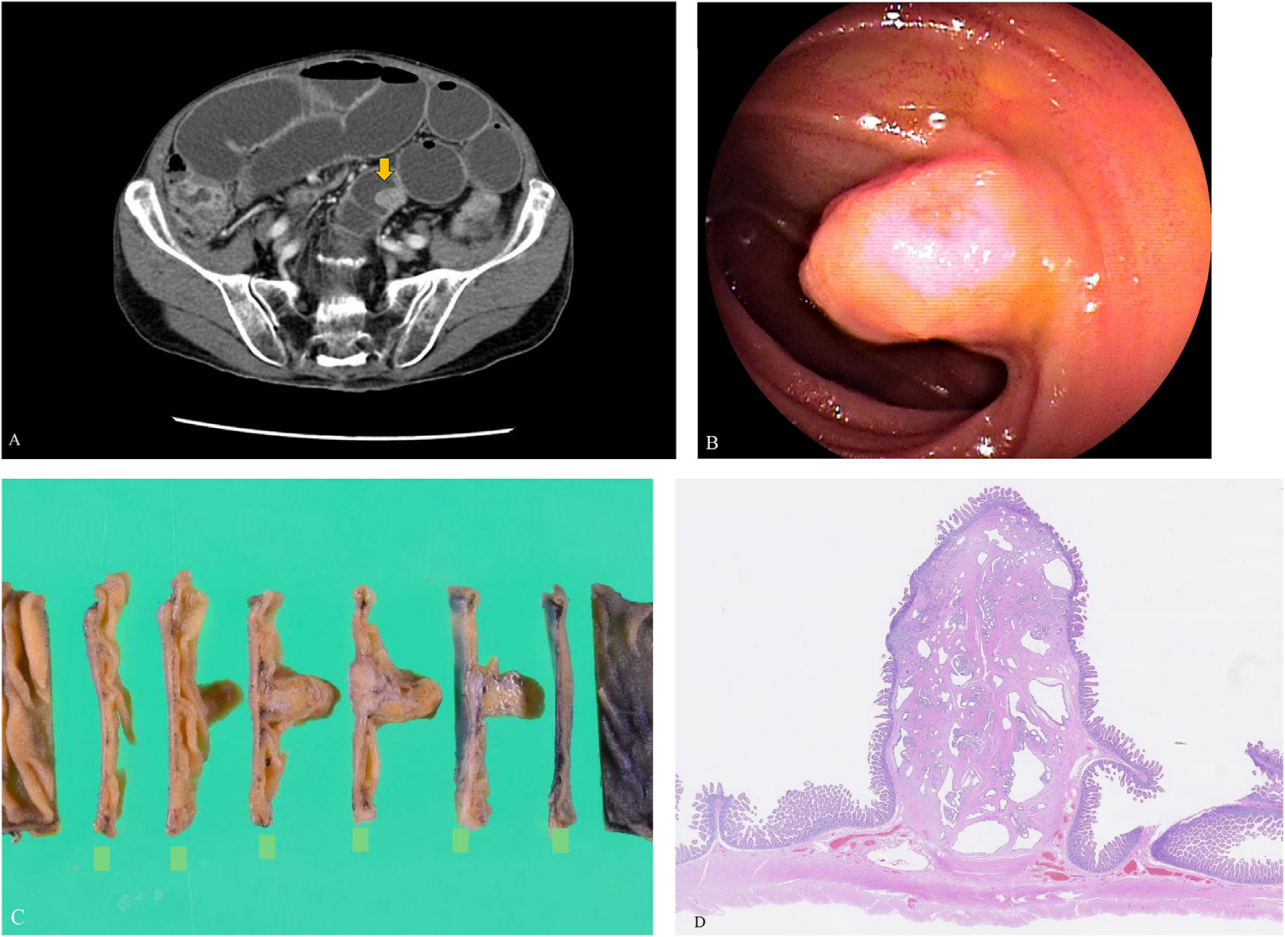# Ectopic Pancreas in the Small Intestine

**DOI:** 10.1016/j.gastha.2023.10.011

**Published:** 2023-10-29

**Authors:** Sachiyo Onishi, Takashi Ibuka, Masahito Shimizu

**Affiliations:** Department of Gastroenterology and Internal Medicine, Gifu University Graduate School of Medicine, Yanagido, Gifu, Japan

A 72-year-old male was diagnosed with intestinal obstruction due to a groin hernia. A CT scan at that time incidentally revealed a small intestinal tumor (Figure A). The patient was asymptomatic, and no abnormal values were pointed out in blood tests, including tumor markers. After the hernia was treated, a 10 mm submucosal tumor was identified in the ileum by double-balloon endoscopy (Figure B). Endoscopic findings suggested a gastrointestinal stromal tumor-like submucosal tumor. A partial resection of the small intestine was then performed (Figure C). Pathology revealed a Heinrich type III ectopic pancreas (Figure D). The patient is currently under outpatient observation.

Isolated ectopic pancreas of the ileum is extremely rare and has not been reported, as ectopic pancreas of the ileum occurs in only 2.5% of cases, and most of them occur within Meckel's diverticulum.

**Figure:**